# Population pharmacokinetic and pharmacodynamic model guided weight-tiered dose of AST-001 in pediatric patients with autism spectrum disorder

**DOI:** 10.3389/fphar.2024.1452526

**Published:** 2024-12-16

**Authors:** Soyoung Lee, Su-Kyeong Hwang, Jung-Sook Cho, Hyung Chul Ryu, Jae-Yong Chung

**Affiliations:** ^1^ College of Pharmacy, Chungnam National University, Daejeon, Republic of Korea; ^2^ Department of Pediatrics, School of Medicine, Kyungpook National University, Daegu, Republic of Korea; ^3^ Astrogen Inc., Daegu, Republic of Korea; ^4^ Department of Clinical Pharmacology and Therapeutics, Seoul National University College of Medicine, Seoul, Republic of Korea; ^5^ Department of Clinical Pharmacology and Therapeutics, Seoul National University Bundang Hospital, Seongnam, Republic of Korea

**Keywords:** Vineland adaptive behavior scales-II, L-serine, autism spectrum disorder, weight-tiered dose, PK-PD (pharamacokinetic-pharmacodynamic) model

## Abstract

AST-001, a novel syrup formulation of L-serine, was developed for the treatment of autism spectrum disorders (ASD) in pediatric patients. This study aimed to establish a pharmacokinetic (PK)-pharmacodynamic (PD) model to elucidate the effect of AST-001 on adaptive behavior in children with ASD. Due to the absence of PK samples in pediatric patients, a previously published population PK model was used to link the PD model by applying an allometric scale to body weight. The time courses of Korean-Vineland Adaptive Behavior Scale-II Adaptive Behavior Composite (K-VABS-II-ABC) scores were best described by an effect compartment model with linear drug effects (Deff, 0.0022 L/μg) and linear progression, where an equilibration half-life to the effect compartment was approximately 15 weeks. Our findings indicated a positive correlation between the baseline K-VABS-II-ABC score (E0, 48.51) and the rate of natural progression (Kprog, 0.015 day^−1^), suggesting enhanced natural behavioral improvements in patients with better baseline adaptive behavior. Moreover, age was identified as a significant covariate for E0 and was incorporated into the model using a power function. Based on our model, the recommended dosing regimens for phase III trials are 2, 4, 6, 10, and 14 g, administered twice daily for weight ranges of 10–13, 14–20, 21–34, 35–49, and >50 kg, respectively. These doses are expected to significantly improve ASD symptoms. This study not only proposes an optimized dosing strategy for AST-001 but also provides valuable insights into the PK-PD relationship in pediatric ASD treatment.

## Introduction

Autism spectrum disorder is characterized by impaired social communication, repetitive behavior, and restricted interests (Sharma et al., 2018). The prevalence of autism is continuously increasing from 62/10,000 in 2012 to 65/10,000 in 2022 (Eom et al., 2023). More than 60% of autism patients have intact cognition and no language impairment, while adaptive functions are deficient (Saulnier and Klaiman, 2022). Because earlier interventions of adaptive behavior in autism patients are strongly correlated with the improvement of autism outcomes in adults (Saulnier and Klaiman, 2022), a novel therapy for improving those adaptive functions is needed.

The Vineland Adaptive Behavior Scale-II (VABS-II) is the adaptive behavior test to assess the individual’s social deficits and developmental retardation (Sparrow et al., 2011). It is widely used to evaluate the developmental progress and adaptive behaviors in individuals, including those with autism spectrum disorder, and assess the efficacy of the treatment (Sparrow et al., 2011; Klin et al., 2007). The standard version of Korea is the K-VINELAND-II and Korean-VABS-II Adaptive Behavior Composite (K-VABS-II-ABC) score. The K-VABS-II-ABC score is the calculated scores (standard scores) from four domains of communication, daily living skills, socialization, and motor skills, where motor skills are additionally evaluated in pediatrics less than the age of seven (Hwang et al., 2015; Oh et al., 2021). Adaptive behavior can be categorized into three classes with VABS-II score: severe impairment, <71; moderate impairment, 71–84; and mild to no impairment, >84 (Weitlauf et al., 2014). Previous research had reported that the minimal clinically significant difference in VABS-II score is 2–3.75 points (Chatham et al., 2018).

The pharmacological treatment of autism includes different classes of atypical antipsychotic drugs, antidepressants, and modulators of the sympathetic and parasympathetic (Aman et al., 2008). However, those medications show highly variable treatment responses between patients and only improve disruptive repetitive behaviors (Sharma et al., 2018). Given concerns of adverse events for pharmacological intervention in the long-term, concomitant non-pharmacological treatment involving music, cognitive-behavioral, and social-behavioral therapy are used to improve social interaction in autism patients (Sharma et al., 2018; Meza et al., 2022). One of the emerging treatment candidates is L-serine which has neuroprotective effects and a prominent safety profile (Ye et al., 2021; Garofalo et al., 2011; Gantner et al., 2019).

In a previous mouse model study, L-serine improved sociability by modulating SK channels to restore dopamine neuron firing in the ventral tegmental area impaired in autism spectrum disorder (Um et al., 2023). Also, L-serine has demonstrated clinical benefits in several neurological and metabolic disorders, such as Alzheimer’s disease, hereditary sensory neuropathy, and GRIN-related disorders, at doses of up to 850 mg/kg/day (Levine et al., 2017; Fridman et al., 2019; Krey et al., 2022; Yulug et al., 2023). These studies reported improvements in symptoms or slowed disease progression, with no significant safety concerns (Krey et al., 2022; Yulug et al., 2023; Julia-Palacios et al., 2024).

AST-001 is an L-serine syrup formulation developed to treat autism spectrum disorders. In a previous study, we evaluated the pharmacokinetic (PK) properties of AST-001 after single and multiple administration in healthy subjects (Lee et al., 2022). Orally administered AST-001 rapidly absorbed with a peak concentration reached in 0.75–2 h and showing a half-life of 7–14 h. Dose-linearity was observed in the range of 10–30 g, and endogenous L-serine production rate was estimated as 287 mg/h in population PK analysis (Lee et al., 2022). Based on the modeling and simulation, we suggested five weight ranges with a fixed-dose regimen that could achieve the target exposure to pediatrics. In a phase II clinical trial, 12 weeks of treatment of AST-001 twice daily improved functional adaptation in pediatric patients with autism (Kim et al., 2024; Kim et al., 2023). The primary efficacy endpoint, 12 weeks K-VABS-II-ABC score change from baseline in the ANOVA model adjusted for age and baseline score was significantly increased in the AST-001 high dose treatment group compared to the placebo group, showing the difference in score change between the two groups (LS mean ± SE) was 1.43 ± 0.69 points, which was statistically significant (P = 0.042) (Kim et al., 2023). As of December 2023, a phase III clinical trial is currently ongoing (KCT0008915) to confirm the efficacy of AST-001.

This study aimed to develop a population PK-PD model to describe changes in K-VABS-II-ABC scores with natural autism progression and their drug effects. As PK samples were not obtained from pediatric patients, the PD model was linked to the adult PK model using an allometric scale. The established PK-PD model was used to determine the weight-tiered efficacious doses of AST-001 in pediatric patients with autism for a phase III trial.

## Methods

### Data

Data from a multi-center, randomized, double-blind, placebo-controlled Phase II trial were used in this analysis (Kim et al., 2024; Kim et al., 2023). This study consisted of two parts: primary and extended treatment periods. The patients were randomly allocated to the placebo, low-dose, or high-dose groups and the respective treatments were administered for 12 weeks during the primary study period. Based on the previous population PK analysis, 2, 4, 7, 10, and 14 g twice daily (BID) fixed-dose regimens for each weight range of 10–14 kg, 15–24, 25–37, 38–51, and 52–60 kg were administered in the high-dose group, which showed similar exposure to 400 mg/kg/day (Lee et al., 2022). Half of those weight-tiered doses were administered for the low-dose group, which was equivalent to 200 mg/kg/day exposure. In the high-dose group, there was a two-week titration period with a low dose followed by treatment with a high dose for the remaining 10 weeks. During the extension treatment period, the low- and high-dose groups continuously received each treatment for an additional 12 weeks, whereas the placebo group received a high dose of AST-001 including the titration period. For follow-up assessment, patients visited the center at 36 weeks, which is 12 weeks after the last dose of AST-001. The K-VABS-II-ABC score was obtained at baseline, week 12, week 24, and week 36 to evaluate the PD response.

The trial was conducted following Korean Good Clinical Practice and the Declaration of Helsinki and was approved by the Ministry of Food and Drug Safety. The study information was registered in the Korean Open Clinical Trial Registry (KCT0007519), and all patients or their legally acceptable representatives provided written informed consent before study enrollment.

### Software

During the initial model development, both NONMEM (version 7.4.0, ICON Development Solutions, Ellicott City, United States) and Monolix^®^ (version 2021R1, Lixoft, Antony, France) software were used for non-linear mixed effects modeling. The final PK/PD modeling was implemented using Monolix^®^ with the stochastic approximation expectation-maximization algorithm. Simulations were run using Simulx^®^ (version 2021R1, Lixoft, Antony, France) and bootstrap was performed using R with Rsmlx package (version 4.0). The virtual pediatric populations were generated using “httk” package in R to incorporate the age-weight correlations in virtual pediatrics.

### Pharmacokinetic model

Due to the absence of PK samples from pediatric patients, the previously published population PK model of AST-001 was used to link the PD model. Briefly summarizing the PK model, a two-compartment model, zero-order absorption, and linear elimination combined with zero-order L-serine production were used (Lee et al., 2022). As we changed the modeling software, we re-estimated the population PK model and confirmed that the parameter estimates and precisions were similar to the previous report. We used the final PK model that excluded endogenous production because endogenous L-serine levels interfered with parameter estimation related to drug effects by externally administered AST-001. By applying the empirical allometry coefficient of 0.75 and 1 in clearance and volume of distribution, respectively, we assumed the PK profile of pediatrics. All PK parameters were fixed using final model estimates.

### Pharmacodynamic model

The K-VABS-II-ABC scores were used to determine the PD response to the drug effect. To link the PK with PD, we used the population PK parameter (PPP) approach. Turnover, effect compartment, and direct models were evaluated to describe the time course of the K-VABS-II-ABC score.

The inter-individual variabilities of PD parameters were explored using a log-normal distribution. The additive error model was used to account for residual unexplained variability in the K-VABS-II-ABC scores. To identify changes in the K-VABS-II-ABC score over time in the placebo group, we explored constant baseline and linear progression models. The AST-001 concentration-response relationship for K-VABS-II-ABC scores was evaluated using the maximum effect (E_max_) and linear drug effect models.

Potential covariates, including age, weight, sex, and baseline total serine level, were evaluated to determine whether they affected PD parameters. Continuous covariates were modeled using a power function normalized to the median of the observations. Covariates were assessed using forward selection (*p* < 0.05), followed by backward elimination (*p* < 0.01). The final covariate model was selected based on mechanistic plausibility and precision of the parameter estimates.

### Model evaluation

The model was evaluated based on both numerical and graphical diagnostics between runs. The model fit was deemed to improve if the OFV decreased by more than 3.84 with one degree of freedom. Goodness-of-fit plots were graphically evaluated by plotting population or individual predictions *versus* observations and inspecting individual weighted residuals over time and individual predictions. Prediction-corrected visual predictive checks were conducted using 500 simulated datasets. Owing to the long run-time, 200 bootstrap runs were conducted to evaluate the precision of the final model estimates. The model was determined to be robust if the median parameter values were similar to the final estimates and the 95% confidence intervals of each parameter were reasonably narrow.

### Simulation

To explore the fixed-dose regimen according to the weight range of the pediatric patients, we simulated the time courses of the K-VABS-II-ABC scores using the final PK/PD model. The 1,000 virtual pediatric demographics were generated for the age range of 2–12 years, with the same proportion of sex enrolled in the study. We then divided the weight ranges of the dataset to regenerate 200 pediatric patients in each treatment group. The simulated scenario included the uncertainty of the population estimated using Monolix.

Because the formulation of the AST-001 syrup was developed as 20 mL/pouch (2 g/20 mL), simulation dose regimens were set to 2, 4, 6, 8, 10, 12, or 14 g twice daily for 12 weeks, considering the convenience for pediatric patients. Various weight ranges and fixed-dose regimens have been explored to achieve target attainment similar to that of the simulated dose regimen in phase II clinical trials. We considered that autism symptoms clinically improved when the K-VABS-II-ABC scores increased by more than two scores at 12 weeks compared with baseline. Thus, we calculated the percentage of virtual pediatric patients who achieved this target. The simulation dose range was established considering the maximum administered dose (560 mg/kg/day) in the phase II trial. Additionally, we compared the dose regimen for the final suggested weight-tiered fixed dose with a weight-based dose of 400 mg/kg/day to confirm that the exposures were similar.

## Results

### Study population

A total of 145 pediatric patients with autism and 570 K-VABS-II-ABC scores were used to develop a PD model. Among them, 193 observations (n = 49), 195 observations (n = 50), and 182 observations (n = 46) were from the placebo, low-dose, and high-dose groups respectively. The study population included 120 males (82.8%) and 25 females (17.2%) aged 2–11 years. The median (min-max) values of weight and baseline total serine were 20.5 (10.7–58.1) kg and 128 (74.8–609) μmol/L, respectively.

### Population pharmacokinetic and pharmacodynamic model

**FIGURE 1:**
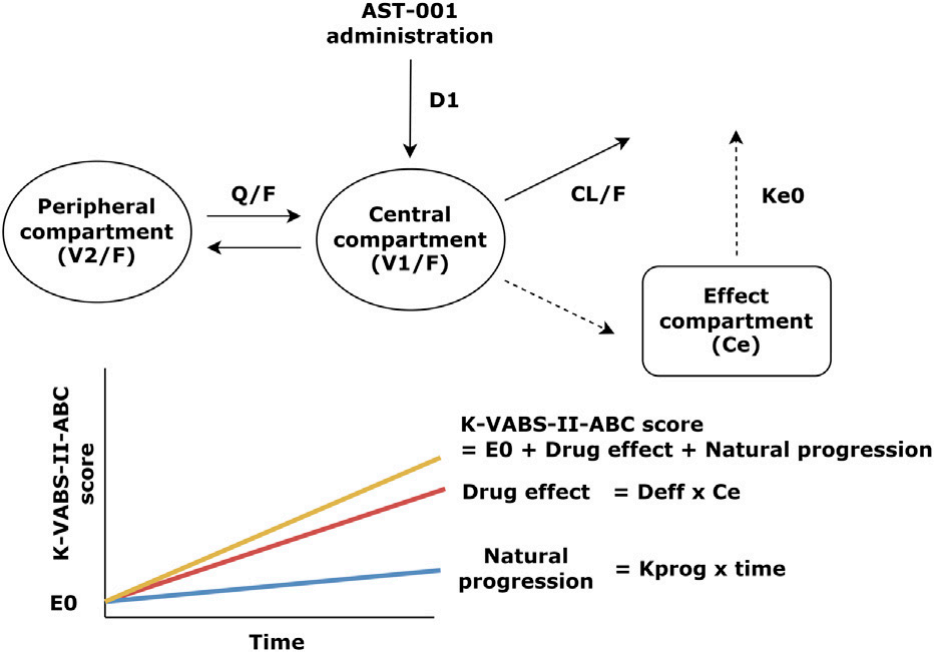
Structure of the population pharmacokinetic and pharmacodynamic model of AST-001 and K-VABS-II-ABC score. D1, duration of zero-order absorption; CL/F, apparent clearance; V1/F, apparent central compartment volume; Q/F, apparent inter-compartmental clearance; V2/F, apparent peripheral compartment volume; ke0, effect-site first order elimination rate constant; E0, baseline VABS score; Deff, linear slope of drug effect regarding effect site concentration; Ce, effect site concentration, Kprog, slope of natural VABS score progression; K-VABS-II-ABC, Korean-Vineland Adaptive Behavior Scale-II Adaptive Behavior Composite.

The final PK-PD models of the AST-001 and K-VABS-II-ABC scores are presented in Figure 1. The PD model is best described as an effect-compartment model with linear drug effects. The goodness-of-fit plot showed an adequate model structure without any apparent misspecifications (Supplementary Figure S1). The prediction-corrected visual predictive check showed that the model generally described the observations well (Supplementary Figure S2). The precision of the parameters was generally acceptable, with relative standard errors (RSEs) of less than 25%, except for the linear slope of the drug effect (Deff). The final estimates were similar to the median bootstrap values and included in the 95% confidence intervals of the bootstrap results, implying that the model was robust and stable (Table 1).

**TABLE 1:** Final parameter estimates of population pharmacodynamic model of K-VABS-II-ABC score.

Parameters	Estimates	RSE (%)	Bootstrap (n = 200)^a^	
			Median	95% CI
*Fixed effects*				
Ke0 (1/day)	0.0065	7.92	0.0070	0.0043–0.010
E0	48.51	1.66	48.48	46.8–50.14
β_age_	−0.21	18.4	−0.22	−0.28 – −0.14
Deff (L/μg)	0.0022	37.5	0.0024	0.0008–0.0046
Kprog (1/day)	0.015	12.0	0.015	0.012–0.019
*Random effects*				
ΩKe0	0.21	22.3	0.25	0.14–0.51
ΩE0	0.2	6.01	0.2	0.17–0.22
ΩDeff	1.4	20.8	1.31	0.89–1.97
ΩKprog	0.018	8.27	0.018	0.014–0.021
*Correlation*				
Correlation E0-Kprog	0.48	16.9	0.49	0.34–0.63
*Residual error*				
additive error	1.6	4.56	1.6	1.39–1.81

^a^ 200 runs (100%) have been converged successfully.

K-VABS-II-ABC, Korean-Vineland Adaptive Behavior Scale-II, adaptive behavior composite; Ke0, effect-site first order elimination rate constant; E0, baseline K-VABS-II-ABC, score; β_age_, exponent of power relationship between normalized age (5 years) and E0; Deff, linear slope of drug effect regarding effect site concentration; Kprog, slope of natural K-VABS-II-ABC, score progression; RSE, residual standard error.

The slope of the natural K-VABS-II-ABC score progression (Kprog) was estimated to be 0.015 day^−1^ with a standard deviation of random effects of 0.018 in a normal eta distribution (Table 1). The Deff was estimated as 0.0022 L/μg and the equilibration half-life to the effect compartment was approximately 15 weeks (Table 1). Age was identified as a significant covariate for the baseline K-VABS-II-ABC score (E0) and was included in the model using a power function. There was a positive correlation between E0 and Kprog, suggesting that patients with poorer adaptive behavior at baseline (lower E0) tend to exhibit less natural improvement in their behavior over time.

### Weight-tiered dose simulation

**FIGURE 2:**
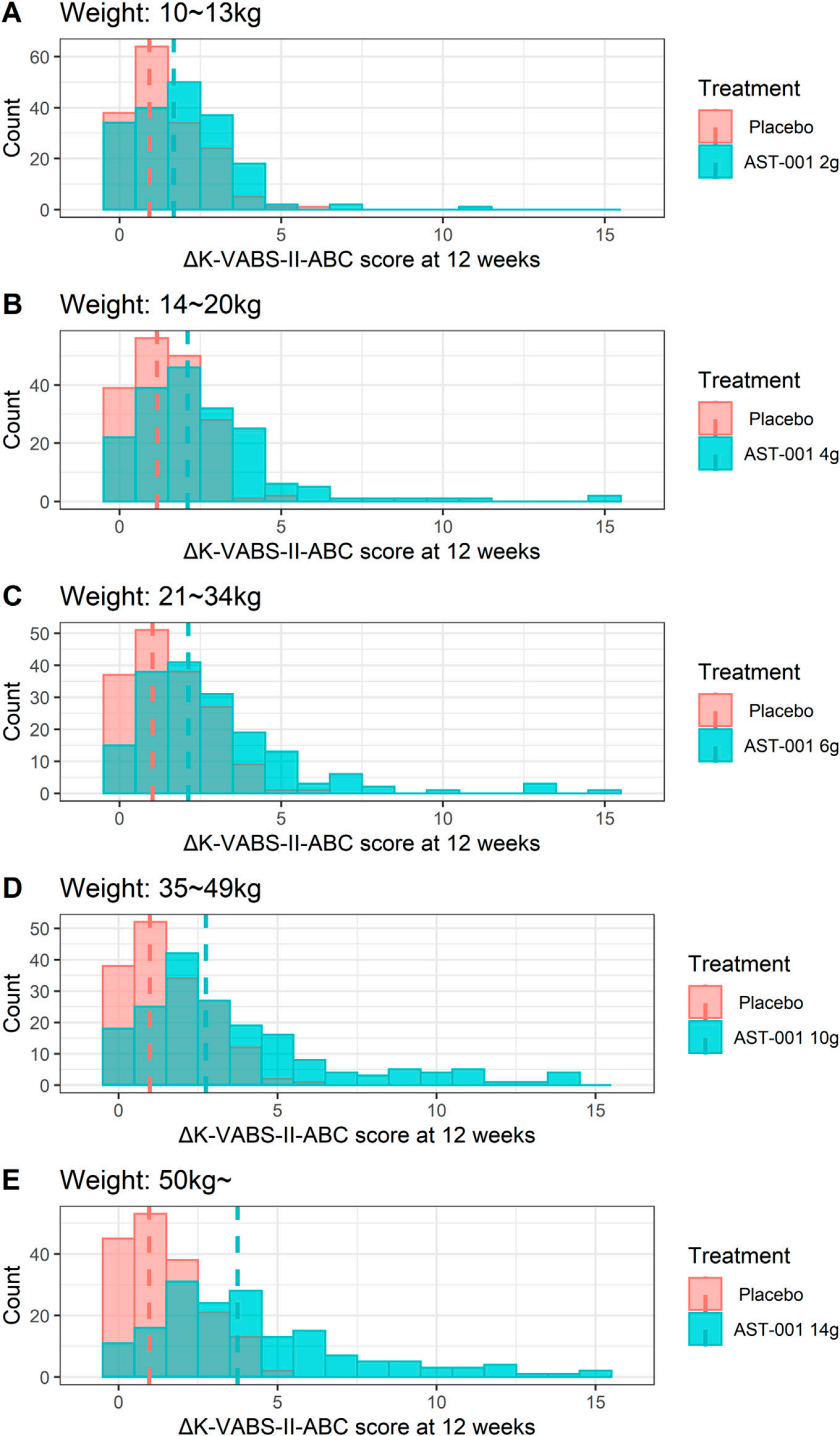
Histogram plots for changes in K-VABS-II-ABC score at 12 weeks from baseline in 200 virtual pediatric patients without AST-001 treatment and after **(A)** 2 g (10–13 kg), **(B)** 4 g (14–20 kg), **(C)** 6 g (21–34 kg), **(D)** 10 g (35–49 kg), and **(E)** 14 g (more than 50 kg) twice daily dose treatment. The dashed line represents the median changes in K-VABS-II-ABC score at 12 weeks in each treatment. K-VABS-II-ABC, Korean-Vineland Adaptive Behavior Scale-II Adaptive Behavior Composite.

Considering the maximum administration dose of 560 mg/kg/day in the phase 2 trial, we established weight-tiered doses of 10–13 kg (2 g BID), 14–20 kg (4 g BID), 21–34 kg (6 g BID), 35–49 kg (10 g BID), and >50 kg (14 g BID) for the simulation. These regimens correspond to 308–400 mg/kg/day, 400–571 mg/kg/day, 353–571 mg/kg, 408–571 mg/kg, and less than 560 mg/kg. Our simulation results showed that 12 weeks of twice-daily administration of AST-001 at 2, 4, 6, 10, and 14 g with each weight band improved K-VABS-II-ABC scores from 1.7 to 3.7 compared to the baseline (Table 2). In contrast, the placebo group showed 0.9 to 1.2 increased K-VABS-II-ABC scores overall. The virtual pediatric population had improved K-VABS-II-ABC scores in the weight-band fixed-dose treatments compared to the placebo group (Figure 2). Target attainment was higher in the high-dose weight band group (Figure 2; Table 2). Although the proportion of participants in the 10–13 kg (2 g BID) group showed a lower target attainment of 40.5%, it was twice as high as that in the placebo group (Table 2). In addition, the proportions of target attainment were 40.5%, 51.5%, 54%, 65%, and 73%, similar to the simulation results of the phase 2 trial weight-band dose (Table 2).

**TABLE 2:** Simulation results of changes in K-VABS-II-ABC score at 12 weeks from baseline according to the weight-tiered dose in placebo group and treatment group with different dose scenario.

Weight band Dose	Placebo				Weight band Dose	Phase III dose scenario				Weight band Dose	Phase II dose scenario			
	ΔK-VABS-II-ABC at 12 weeks^a^			Target attainment (%)^b^		ΔK-VABS-II-ABC at 12 weeks^a^			Target attainment (%)^b^		ΔK-VABS-II-ABC at 12 weeks^a^			Target attainment (%)^b^
	5%	50%	95%			5%	50%	95%			5%	50%	95%	
10 ∼ 13 kg (2 g BID)	−1.75	0.92	3.34	19.5	10 ∼ 13 kg (2 g BID)	−0.8	1.67	4.01	40.5	10 ∼ 14 kg (2 g BID)	−0.8	1.67	4.01	40.5
14 ∼ 20 kg (4 g BID)	−1.06	1.15	3.3	29	14 ∼ 20 kg (4 g BID)	−0.71	2.06	7.65	51.5	15 ∼ 24 kg (4 g BID)	−0.7	2.11	7.72	53
21 ∼ 34 kg (6 g BID)	−1.69	1.02	3.74	29	21 ∼ 34 kg (6 g BID)	−0.91	2.13	13.05	54	25 ∼ 37 kg (7 g BID)	−0.9	2.32	15.4	56
35 ∼ 49 kg (10 g BID)	−1.56	0.98	3.78	29.5	35 ∼ 49 kg (10 g BID)	−0.07	2.77	20.99	65	38 ∼ 51 kg (10 g BID)	−0.07	2.76	20.99	65
50 kg ∼ (14 g BID)	−1.57	0.96	3.65	27	50 kg ∼ (14 g BID)	−0.65	3.74	24.81	73	52 kg ∼ (14 g BID)	−0.67	3.73	24.76	73

^a^ Changes in K-VABS-II-ABC score at 12 weeks from baseline.

^b^ The percentage of pediatric patients with an increase of more than 2-scores in the changes in K-VABS-II-ABC, score at 12 weeks from baseline.

K-VABS-II-ABC, Korean-Vineland Adaptive Behavior Scale-II, adaptive behavior composite.

**FIGURE 3:**
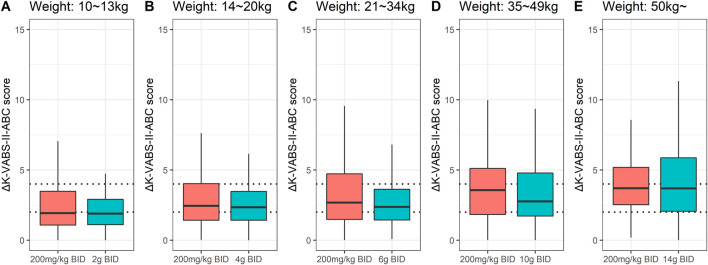
Box plots for changes in K-VABS-II-ABC score at 12 weeks from baseline (ΔK-VABS-II-ABC) in 200 virtual pediatric patients after AST-001 treatment with 200 mg/kg twice daily (BID) regimen and **(A)** 2 g BID (10–13 kg), **(B)** 4 g BID (14–20 kg), **(C)** 6 g BID (21–34 kg), **(D)** 10 g BID (35–49 kg), and **(E)** 14 g BID (more than 50 kg) in each weight ranges. The dotted line represents the ΔK-VABS-II-ABC score of 2 and 4. K-VABS-II-ABC, Korean-Vineland Adaptive Behavior Scale-II Adaptive Behavior Composite.

The suggested final weight-tiered fixed dose was compared with 200 mg/kg twice daily for each weight range in the pediatric population. Overall, the two regimens showed similar improvements in K-VABS-II-ABC scores (Figure 3). Although the 200 mg/kg twice daily regimen in the 21–34 kg and 35–49 kg weight groups led to greater median score improvements of 20% and 31% respectively, the increases were not statistically significant.

**FIGURE 4:**
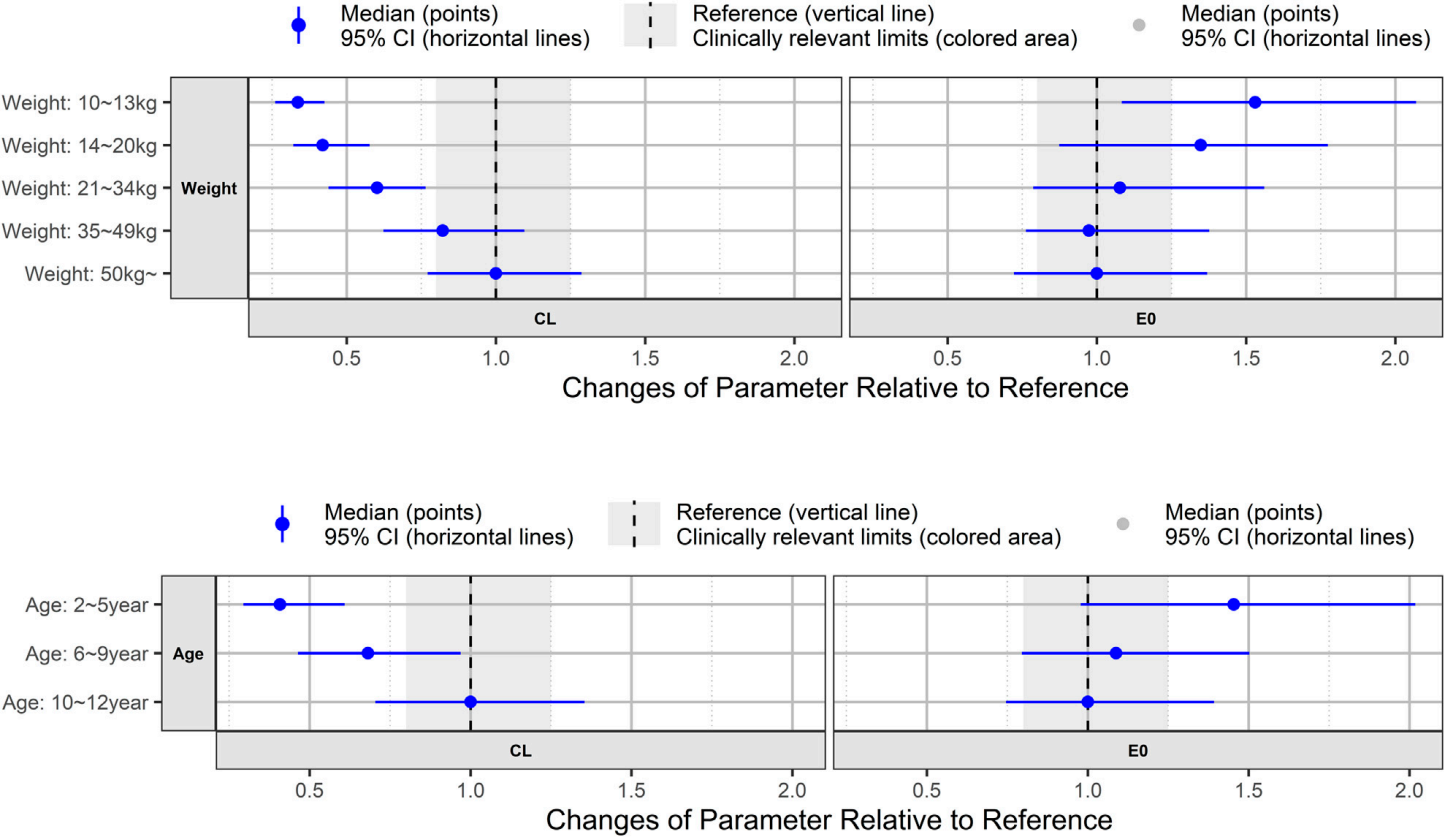
Forest plot showing age and weight effect on model-predicted apparent clearance (CL) of AST-001 and baseline K-VABS-II-ABC score (E0). The blue symbols represent the median model predicted CL and E0 ratio, and the whiskers represent the 95% confidence interval. K-VABS-II-ABC, Korean-Vineland Adaptive Behavior Scale-II Adaptive Behavior Composite.

To explore the effects of covariates on apparent clearance (CL/F) and baseline K-VABS-II-ABC scores in virtual pediatric patients, we analyzed forest plots across different weight and age range categories. The CL/F of AST-001 was 34% lower in the lower-weight group (10–13 kg) than in the group weighing >50 kg (Figure 4). As age and weight were positively correlated, the 2–5 years age group exhibited a 41% decrease in CL/F compared to the 10–12 years age group. Pediatric patients weighing more than 21 kg showed relatively comparable E0 values, whereas those weighing less than 20 kg displayed a 35%–53% increase in E0. Similarly, the 6–12 years age group showed comparable E0 values, whereas the 2–5 years age group demonstrated a 45% increase in E0 (Figure 4).

## Discussion

In this study, we explored the effects of L-serine in pediatric patients with autism spectrum disorders using a population PK-PD model approach. Based on our previously published PK model, we linked PD with the K-VABS-II-ABC scores using an effect-compartment model. The increase in K-VABS-II-ABC scores due to AST-001 treatment was characterized by a linear relationship with the concentration at the effect site. Additionally, we incorporated a linear progression model to describe the changes in K-VABS-II-ABC scores observed in the placebo group.

In the Phase II clinical trial, the least-squares mean change from baseline in K-VABS-II-ABC scores at 12 weeks was 2.82 ± 0.50 for the low-dose group and 1.73 ± 0.51 for the placebo group (Kim et al., 2024). In comparison, the high-dose and placebo groups showed corresponding changes of 3.08 ± 0.49 and 1.66 ± 0.48, respectively (Kim et al., 2024). The mean changes in K-VABS-II-ABC scores at 12 weeks from baseline were significantly higher in the high-dose group than in the placebo group, showing the difference in score change between the two groups was 1.43 ± 0.69 points (*p* = 0.042) (Kim et al., 2024; Kim et al., 2023). These results were also well predicted by our model, in which the high-dose group showed an increase in K-VABS-II-ABC scores ranging from 1.67 to 3.73. Similarly, significant improvements were observed in the Communication and Motor Skills domains of the K-VABS-II in the high-dose group, while Clinical Global Impression-Severity scores were significantly reduced in both dose groups (Kim et al., 2024).

In our model analysis, age was identified as a significant covariate affecting baseline K-VABS-II-ABC scores. Consistent with a previous report (Klin et al., 2007), a negative correlation between age and baseline K-VABS-II-ABC score was also observed in our study (Pearson correlation coefficient = −0.2793, *p* = 0.0007). As shown in the simulation results (Figure 4), the 6–12 years age group showed comparable E0 values, whereas the 2–5 years age group exhibited a 45% increase in the baseline score. Given that the K-VABS-II-ABC score includes motor skills assessments (Hwang et al., 2015), particularly in children younger than 7 years, it is plausible that higher scores are observed in the younger age group. Therefore, we established the efficacy end point as the change in scores at 12 weeks from the baseline.

Our study is the first to report a population PK-PD model linking L-serine treatment with K-VABS-II-ABC scores in pediatric patients with autism spectrum disorder. Patients with higher baseline values exhibited a greater response to L-serine treatment and a better prognosis for adaptive behavior. In our virtual fixed-dose regimen simulation, the group with E0 less than 45 demonstrated a median Deff of 1.45 (mL/μg) and a kprog of 0.06 (week^−1^). Conversely, groups with higher baselines exhibited more pronounced drug effects and score progression, with a median Deff of 1.65 (mL/μg) and kprog of 0.14 (week^−1^). This finding is consistent with Phase II results, where pediatric patients aged 2–6 years with higher baseline scores demonstrated significant improvements following AST-001 treatment (Kim et al., 2024).

Currently, we are developing an AST-001 syrup formulation containing 2 g of L-serine in each 20 mL pouch. Considering convenience for pediatric patients, we developed a syrup formulation. The phase III dose was selected based on the doses provided in these pouches to give accurate doses in individuals. Several weights and dose ranges were investigated using the final model. The optimal weight-band doses determined to achieve sufficient improvement in adaptive behavior were 10–13 kg (2 g BID), 14–20 kg (4 g BID), 21–34 kg (6 g BID), 35–49 kg (10 g BID), and >50 kg (14 g BID). These regimens improved the K-VABS-II-ABC score by more than 2 points overall compared to the placebo group, and their effects were similar to those of the 200 mg/kg BID dose regimen. By applying a weight-tiered fixed-dose regimen, we could offer a convenient dosing option for pediatric patients, along with effective treatment results.

A limitation of our study is that we hypothesized the PK in pediatric patients using empirical allometry scaling. Although AST-001 has a linear PK profile with a relatively short half-life in healthy subjects, patients with autism may have different PK characteristics. In addition, the adjustments to the Phase III weight-tiered dosing regimen were based on formulation constraints and dosing convenience considerations, which were not fully addressed during the transition from Phase I to Phase II study. Further phase III study data, including L-serine trough concentration and efficacy data, can provide a more elaborate exposure-response relationship.

## Conclusion

We developed a population PK-PD model of externally administered L-serine and described the time course of the K-VABS-II-ABC score and drug effects for up to 36 weeks. The K-VABS-II-ABC scores increased with increasing L-serine concentration at the effect site. The baseline score decreased with increasing age in pediatric patients with autism, and a higher baseline score was associated with a better response to L-serine treatment. We suggest 2, 4, 6, 10, and 14 g twice-daily regimens for the 10–13, 14–20, 21–34, 35–49, and >50 kg weight ranges, respectively, which are expected to improve autism symptoms in ongoing phase III trial.

## Data Availability

The datasets presented in this article are not readily available because of the confidentiality of clinical trial data. Requests to access the datasets should be directed to Su-Kyeong Hwang, skhwang@astrogen.co.kr.
