# Supplementary material for: Population pharmacokinetic and pharmacodynamic model guided weight-tiered dose of AST-001 in pediatric patients with autism spectrum disorder
Source: Front Pharmacol. 2024 Dec 16;15:1452526. doi: 10.3389/fphar.2024.1452526 (PMC11682956; doi:10.3389/fphar.2024.1452526)
Supplement: Supplementary file 1 [file Image1.pdf]

SUPPLEMENTARY MATERIALS

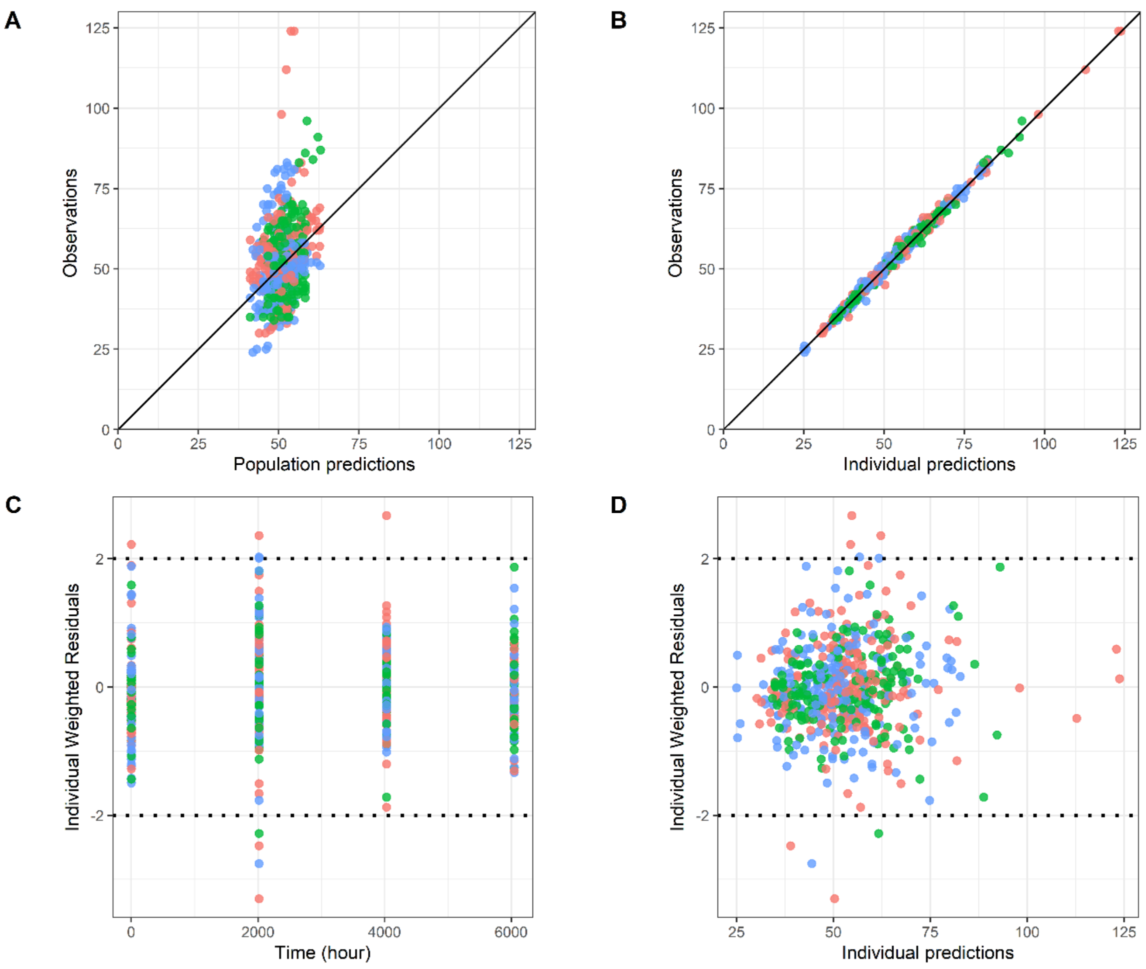

Figure S1. Goodness-of-fit plots for the final model. (A) Population prediction versus observation of K-VABS-II-ABC score, (B) individual prediction versus observation of K-VABS-II-ABC score (C) time versus individual weighted residuals, and (D) individual prediction of K-VABS-II-ABC score versus individual weighted residuals. The red, green, and blue dots represent the treatment group of 200 mg/kg/day, 400 mg/kg/day, and placebo then 400 mg/kg/day, respectively.

K-VABS-II-ABC, Korean-Vineland Adaptive Behavior Scale-II Adaptive Behavior Composite

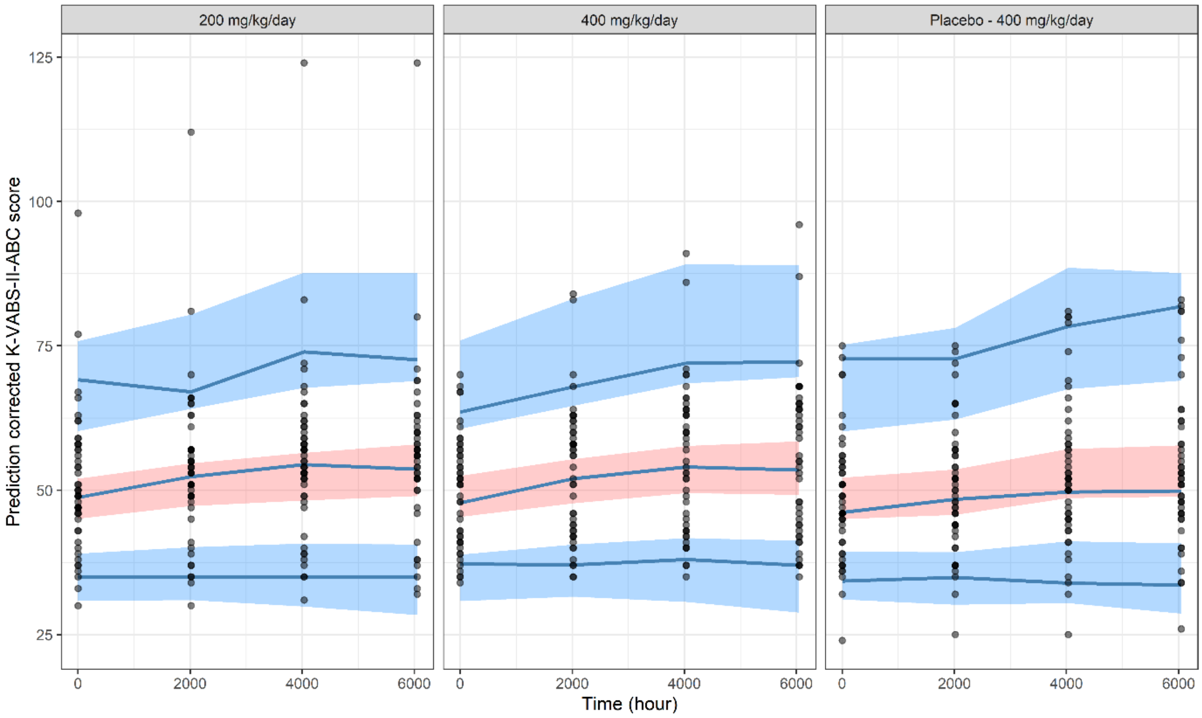

Figure S2. Prediction corrected K-VABS-II-ABC score-time profiles in each treatment groups. The blue lines represent the observed 5th, 50th, and 95th percentiles. The red shaded area represents the 95% confidence interval of the simulated 50th percentile, and the blue shaded area represents the corresponding simulated 5th and 95th percentiles. The black dots represent the observed values.

K-VABS-II-ABC, Korean-Vineland Adaptive Behavior Scale-II Adaptive Behavior Composite
